# Consumer insights from a feasibility study on remote and extended use of a novel non-invasive wearable fetal electrocardiogram monitor

**DOI:** 10.1038/s41746-025-01628-9

**Published:** 2025-04-21

**Authors:** Debjyoti Karmakar, Tarini Paul, Emerson Keenan, Marimuthu Palaniswami, Kaitlin Constable, Erica Spessot, Fiona Brownfoot

**Affiliations:** 1 University of Melbourne, Mercy Hospital for Women, Melbourne, VIC Australia; 2 Department of Obstetrics and Gynaecology, Mercy Hospital for Women, Melbourne, VIC Australia; 3 Department of Electrical Engineering, The University of Melbourne, Melbourne, VIC Australia; 4 Epworth Freemasons, Melbourne, VIC Australia

**Keywords:** Translational research, Outcomes research

## Abstract

The COVID-19 pandemic accelerated the adoption of telehealth and remote monitoring in obstetric care. This study assessed pregnant patients’ perceptions before and after using a novel non-invasive fetal electrocardiogram (NI-FECG) device. The trial is prospectively registered on the Australia New Zealand Clinical Trials Registry (ANZCTRN12621001260819; submitted June 9th, 2021; approved September 17th, 2021). Seventy participants from 36 weeks’ gestation completed pre- and post-use surveys. Interest in continuous and home fetal monitoring was high (79% and 90%, respectively). Post-use, 89% reported satisfaction; over 90% comfortable wearing and removing the sensor. Extended use was acceptable to 76%, and only 3% reported high skin irritation. Sentiment analysis highlighted themes of reassurance, convenience, and reduced anxiety. Some suggested smaller, wireless design. Analysis by natural language processing and clustering provided deeper insights. Findings support strong interest in at-home fetal monitoring; further refinement and education are needed to enhance acceptability. Future research should assess long-term effects on anxiety and clinical outcomes.

## Introduction

The COVID-19 pandemic rapidly transformed perceptions of healthcare delivery by accelerating the innovation and implementation of telehealth across various fields, including obstetrics^1–3^. Telemonitoring has emerged as a safe and effective alternative to hospital admission for managing complicated pregnancies, reducing the need for in-person visits and enhancing patient satisfaction^1,4^. Digital health technologies, such as smartphones, wearables, and health apps, have become increasingly popular among pregnant women, offering new ways to support maternal and fetal health^3–5^. Continuous fetal monitoring, which includes evaluation of fetal wellbeing over days to weeks, provides a safe, convenient, and cost-effective alternative to frequent outpatient care^6,7^. Home monitoring is associated with improved patient-centered care and autonomy and may lead to better perinatal outcomes compared to routine hospital care^2,4^.

Before the COVID-19 pandemic, remote monitoring in pregnancy faced skepticism due to limited experience and concerns about safety and effectiveness. While pre-pandemic studies showed consumer interest, real-world adoption was hindered by regulatory hesitancy, clinician skepticism, and the lack of large-scale validation. The pandemic, however, accelerated acceptance, fostering a more favorable view of remote monitoring, including continuous fetal monitoring^1,2,4^. With advancing technology and increased familiarity, this presents an opportunity to assess whether pregnant women now have a deeper understanding and more positive perceptions of these technologies.

This feasibility study aimed to assess pregnant patients’ perceptions of a novel, wearable, non-invasive fetal electrocardiogram (NI-FECG) device for continuous fetal monitoring at home. We conducted pre- and post-use surveys to evaluate their interest in continuous fetal monitoring, its impact on reassurance and anxiety, and overall acceptance of the device, as understanding these factors is critical for successful integration into prenatal care. Advanced analytics were used to gain deeper insights into user experience, guiding future improvements in device design and integration into prenatal care.

## Results

**Fig. 1:**
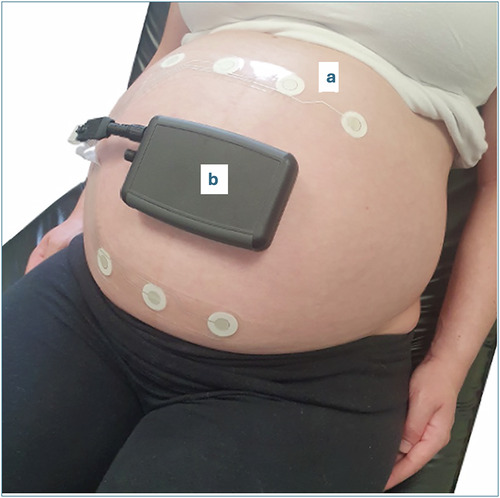
Research grade device used in this trial. This figure presents the research-grade device used in the study, consisting of a wearable sensor patch (**a**) and a hardware unit (**b**) for acquiring electrophysiological data. The current iteration of the commercial-grade device can be accessed at https://www.kalihealthcare.com/.

The study participants had a mean age of 32.3 years and a median BMI of 26.6. Forty-three percent were nulliparous. None reported term stillbirths or neonatal deaths, but 40% had experienced a previous perinatal loss. Stratification of loss types revealed 25 first-trimester losses, two second-trimester losses, and three terminations due to congenital anomalies or genetic conditions. No prior third-trimester losses were reported. Some participants experienced multiple types of loss in the past. Histories of anxiety and depression were noted in 21% and 11% of participants, respectively. Baseline demographic and clinical details are presented in Table 1*and supplementary information*. Figure 1 depicts the research-grade device used in this study.

**Table 1:** Baseline characteristics of participants in the study

Characteristic	*N* = 70
Participant Age (years), Mean (SD)	32.3 (5.2)
Gestation (weeks), Mean (SD)	38.1 (1.5)
Parity,Median (IQR)	1 (1)
*Nulliparous,Count (percentage)*	30 (43%)
*Multiparous, Count (percentage)*	40 (57%)
BMI, Median(IQR)	26.6(5.8)
BMI category	
*Not specified*	1 (1%)
*Normal*	21 (30%)
*Overweight*	31 (44%)
*Obese*	17 (24%)
Country of birth	See Supplementary Table 1
Occupation	See Supplementary Table 1
Indication of monitoring, Count(percentage)	
*Decreased Fetal Movements*	*23 (33%)*
*Hypertension in Pregnancy*	*13 (19%)*
*Intra-Uterine Growth Restriction(IUGR)*	*5 (7.1%)*
*Gestational Diabetes*	*4 (6%)*
*Post Term/Dates*	*9 (13%)*
*Other*	*16 (23%)*
Smoker, Count(percentage)	
*No*	56 (80%)
*Cease pre-pregnancy*	11 (16%)
*Ceased in pregnancy*	3 (4%)
Mode of previous birth, Count(percentage)	
*No prior births*	28 (40%)
*No prior instrumental birth/Emergency Cesarean (NVBs only)*	14 (20%)
*Prior elective Cesareans only*	1 (1%)
*Prior instrumental births but no Emergency Cesarean*	26 (37%)
*Prior emergency Cesareans but not instrumental*	1 (1%)
History of Anxiety, Count(percentage)	
*No*	55 (79%)
*Yes*	15 (21%)
History of Depression, Count(percentage)	
*No*	62 (89%)
*Yes*	8 (11%)
History of PTSD, Count(percentage)	
*No*	69 (99%)
*Yes*	1 (1%)
Tocophobia, Count(percentage)	
*No*	69 (99%)
*Yes*	1 (1%)
History of postnatal anxiety/depression, Count(percentage)	
*No*	68 (97%)
*Yes*	2 (3%)
History of Previous Perinatal Loss, Count(percentage)	
*No*	42 (60%)
*Yes*	28 (40%)
History of perinatal psychological trauma, Count(percentage)	
*No*	63 (90%)
*Yes*	7 (10%)
History of anatomical birth trauma, Count(percentage)	
*None*	59 (84%)
*Significant PPH*	11 (16%)

### Pre-use survey

**Fig. 2:**
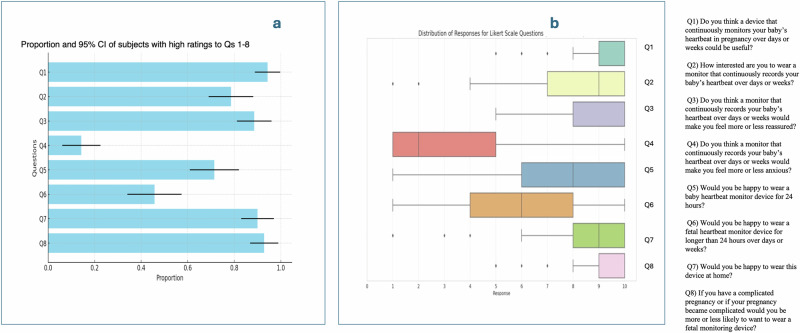
Summary of participants’ responses to pre-use questionnaire. This figure illustrates the distribution of participant responses to the study questionnaires. **a** shows the proportions and 95% confidence intervals (CI) of high-score responders to the pre-use questionnaire. **b** presents the distribution of participant responses to the pre-use questionnaire.

In the “interest and usefulness of continuous fetal monitoring” domain, 94% of patients rated continuous fetal monitoring as highly useful (Likert score ≥7), with a median score of 10 (IQR 1). When asked about their interest in wearing a continuous monitor over days or weeks, 79% provided a high rating (Likert score ≥7). Additionally, 93% rated wearing the device during a complicated pregnancy highly (Likert score ≥7), with a median score of 10 (IQR 1) *(*Fig. 2, Table 2*)*.

**Table 2:** Summary of responses of participants to the PRE-USE and post-use QUESTIONNAIRES

PRE-USE QUESTIONNAIRE (*N* = 70)		
Question	Measure	Value
**Q1: Do you think a device that continuously monitors your baby’s heartbeat in pregnancy over days or weeks could be useful?**	Median(IQR)^a^	10.0, (1.0)
*Low score in the dataset*	Count(Percentage)	4 (6%)
*High score in the dataset*	Count(Percentage)	66 (94%)
	95% CI of Proportion of high score	89–100%
**Q2: How interested are you to wear a monitor that continuously records your baby’s heartbeat over days or weeks?**		9.0 (3.0)
*Low score in the dataset*	Count(Percentage)	15 (21%)
*High score in the dataset*	Count(Percentage)	55 (79%)
	95% CI of Proportion of high score	69–88%
**Q3: Do you think a monitor that continuously records your baby’s heartbeat over days or weeks would make you feel more or less reassured?**		10.0 (2.0)
*Low score in dataset*	Count(Percentage)	8 (11%)
*High score in the dataset*	Count(Percentage)	62 (89%)
	95% CI of Proportion of high score	81–96%
**Q4: Do you think a monitor that continuously records your baby’s heartbeat over days or weeks would make you feel more or less anxious?**		2.0 (4.0)
*Low score in dataset*	Count(Percentage)	60 (86%)
*High score in dataset*	Count(Percentage)	10 (14%)
	95% CI of Proportion of high score	6–23%
**Q5: Would you be happy to wear a baby heartbeat monitor device for 24** **h?**		8.0 (4.0)
*Low score in dataset*	Count(Percentage)	20 (29%)
*High score in dataset*	Count(Percentage)	50 (71%)
	95% CI of Proportion of high score	61–82%
**Q6: Would you be happy to wear a fetal heartbeat monitor device for longer than 24** **h over days or weeks?**		6.0 (4.0)
*Low score in dataset*	Count(Percentage)	38 (54%)
*High score in the dataset*	Count(Percentage)	32 (46%)
	95% CI of Proportion of high score	34–57%
**Q7: Would you be happy to wear this device at home?**		9.0 (2.0)
*Low score in dataset*	Count(Percentage)	7 (10%)
*High score in dataset*	Count(Percentage)	63 (90%)
	95% CI of Proportion of high score	83–97%
**Q8: If you have a complicated pregnancy or if your pregnancy became complicated would you be more or less likely to want to wear a fetal monitoring device?**		10.0 (1.0)
*Low score in dataset*	Count(Percentage)	5 (7%)
*High score in dataset*	Count(Percentage)	65 (93%)
	95% CI of Proportion of high score	87–99%
POST USE QUESTIONNAIRE (*N* = 70)		
**Question**	**Measure**	**Value**
Comfort of Wearing the Device	Median(IQR)^a^	9.0 (2.0)
Comfort of Removing the Sensor Patch	Median(IQR)^a^	8.0 (2.8)
Skin Irritation	Median(IQR)^a^	1.0 (2.0)
Willingness to Wear the Device for 24 h or Longer	Median(IQR)^a^	8.0 (2.8)
Preference for the New Device Over CTG	Median(IQR)^a^	5.0 (2.0)
Overall Satisfaction with the Device	Median(IQR)^a^	9.0 (2.8)

NB: As per convention, a Likert score of ≥7 was considered as high.

^a^Data was non-parametrically distributed.

In the “anxiety and reassurance toward continuous fetal monitoring” domain, 89% of participants reported a high rating (Likert score ≥7) for feeling more reassured with continuous fetal monitoring over days or weeks, with a median score of 10 (IQR 2). Free-text responses indicated that continuous monitoring provided reassurance between scheduled reviews and during periods of decreased fetal movements, with patients feeling more informed about their baby’s wellbeing compared to routine monitoring visits. Only 14% (*n* =10) rated continuous fetal monitoring as making them more anxious (Likert score ≥ 7), with a median score of 2 (IQR 4). Many participants reported reduced anxiety due to the convenience and ability to monitor their baby’s status between scheduled hospital visits.

In the “acceptability of prolonged monitoring” domain, 71% of participants were willing to wear a continuous fetal monitoring device for 24 h (Likert score ≥7), with participants expressing a desire for extra knowledge and confidence from prolonged monitoring. Only 6% (*n* = 4) rated this option extremely low (Likert score 1), citing concerns about device comfort, potential restriction during daily activities, and safety during extended use. When asked about wearing the device for longer than 24 hours over days or weeks, 46% gave a high rating (Likert score ≥7), with a median score of 6 (IQR 4). Ten percent were very happy to wear the device for a prolonged period (Likert score 10), while 7% were not happy (Likert score 1), citing concerns about discomfort and interference with daily activities such as showering and sleeping.

Natural language processing analysis of pre-use free text responses to Q3-9 was used to generate word clouds. These showed key themes such as reassurance, convenience, and ease of monitoring. Words like “reassuring,” “less anxious,” and “convenient” were frequently mentioned, while terms like “worry” and “cumbersome” appeared less frequently. Sentiment analysis revealed that questions Q3 (reassurance with continuous fetal monitoring), Q7 (happy to wear the device at home), and Q8 (willingness to wear device during a complicated pregnancy) had higher average positive sentiment, while questions Q4 (anxiety from continuous fetal monitoring) and Q6 (wearing the device over days or weeks) exhibited more neutral or mixed sentiments (please see supplementary information).

Pre-use Cluster analysis divided participants into four groups based on binary responses (Likert score ≥7), sentiment analysis of open-ended feedback, and clinical, demographic, and mental health characteristics (please see supplementary information). Each cluster exhibited distinct perceptions and profiles, highlighting key features that could guide personalized clinical care and interventions. The “Neutral Expectation Group” had a predominantly neutral sentiment with mixed reactions regarding anxiety and at-home monitoring. About 50% showed favorable attitudes towards at-home monitoring. The “Cautiously Interested Diverse Group” displayed diverse viewpoints, predominantly neutral sentiment toward anxiety with continuous monitoring, but showed positive consensus and broad approval for at-home monitoring. This cluster had a higher prevalence of hypertension in pregnancy, greater parity, and was primarily composed of participants from the Oceania-Antarctica region, with a significant proportion engaged in “Home duties.” The “Optimistic Group” had generally favorable attitudes, with strong positive sentiment toward continuous and at-home monitoring; 100% reported positive responses to at-home monitoring. Participants in this cluster showed high satisfaction overall.

The “Mixed Positive Group” leaned towards neutral-positive sentiments regarding anxiety and continuous monitoring, with a positive but varied response to at-home monitoring. This cluster exhibited the highest birth country diversity.

Significant features influencing cluster assignments included variability in anxiety and depression history, BMI, gestational age (predominantly 38–39 weeks), monitoring indications, maternal age (mostly late twenties to early thirties), and occupational background. No clusters reported a high negative mental health impact from continuous monitoring, suggesting a general acceptance across diverse demographic and clinical profiles. “Optimistic Group” and “Mixed Positive Group” clusters demonstrated the highest acceptance (100% favorable ratings) for at-home monitoring.

### Post-use survey

In the “comfort and wearability of the device” domain, 90% of participants reported high comfort while wearing the device (Q1), with 30% rating it as most comfortable (Likert score 10). The comfort of removing the sensor patch was also rated highly by 91% (Q2), with the highest ratings at 7, 10, and 8. Skin irritation was minimal, with only 3% (*n* = 2) reporting high irritation (Q3), and 54% reporting no irritation (rated 1) (Table 2).

In the “willingness to wear the device long-term” domain, 75% of participants were willing to wear the device for 24 h or longer (Q4), with the highest ratings at 10, 9, and 8. The preference for the new device over CTG (Q5) was high for 30% of participants, while 60% rated it neutrally (score of 5), indicating a general comfort with both devices. Overall satisfaction with the device (Q6) was high among 89% of participants, with top ratings at 9 and 10.

**Fig. 3:**
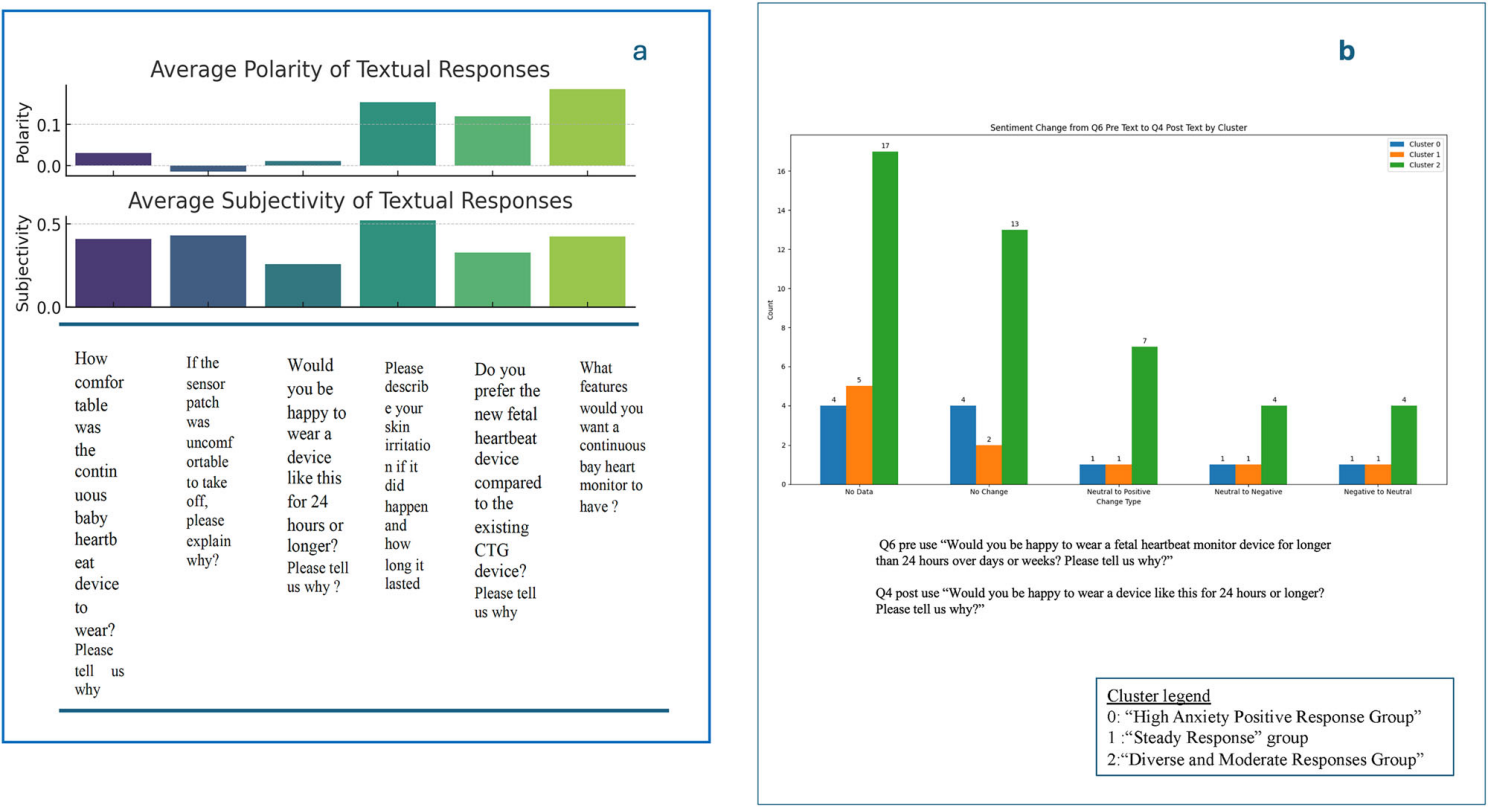
Natural language processing insights on post-use questionnaire responses. This figure provides NLP-based sentiment analysis of participant responses. **a** depicts polarity and subjectivity scores of textual responses to the various post-use questions. **b** shows sentiment changes from pre-use to post-use regarding willingness to use the device beyond 24 hours.

Natural language processing analysis of post-use text responses by word clouds and sentiment analysis (please see supplementary information) yielded themes that include “comfortable,” “bulky,” and “fit” for wearability (Q1a) and “sticky,” “irritation,” and “easy” for removing the sensor patch (Q2a). Positive sentiment was observed for questions related to extended use (Q4a), preference for the new device over CTG (Q5a), and desired features in future iterations (Q7). Sentiment-polarity analysis (please see supplementary information) indicated varied reactions, ranging from slightly negative for ease of removing the sensor patch (−0.014 for Q2a) to moderately positive for desired features (0.185 for Q7), with moderate overall subjectivity (Fig. 3).

Analysis of change in participant perception pre-use vs. post-use is described in supplementary information. In “Usefulness vs. Satisfaction” (PreQ1 vs. PostQ6), the median satisfaction post-use was slightly lower than the median expected usefulness pre-use, decreasing from 10 to 9 (*p* < 0.05). In “Interest vs. Comfort” (PreQ2 vs. PostQ1), no significant change was observed in median scores, with consistent interest and comfort ratings pre- and post-use (*p* > 0.05). In “Willingness to Wear” (PreQ6 vs. PostQ4), a significant increase in ‘high’ ratings from pre-use to post-use was observed, indicating more participants were willing to wear the device for prolonged periods post-use (*p* < 0.01) (Fig. 3).

Cluster analysis of participant perceptions post use (please see supplementary information) generated three distinct groups. “High Anxiety Positive Response Group” comprised 11 participants with a high prevalence of anxiety (10) and depression (8). Post-use ratings were consistently high, with significant positive changes observed in the scores of aligned pre- and post-use questions. “Steady Response Group” comprised 11 participants with the highest average maternal age (34.0 years) and lowest anxiety and depression counts. Minimal changes in perception from pre- to post-use were noted. “Diverse and Moderate Responses Group” was the largest cluster, comprising 48 participants, with moderate anxiety counts (4). A range of sentiment shifts was observed, indicating varied participant perceptions across different domains.

Changes in scores for aligned question pairs were stratified by clusters (please see supplementary information). For “Usefulness vs. Satisfaction” (PreQ1 vs. PostQ6), significant positive changes were noted in the “High Anxiety Positive Response” and “Diverse and Moderate Responses” groups, while the “Steady Response” group showed less improvement. For “Interest vs. Comfort” (PreQ2 vs. PostQ1), the “High Anxiety Positive Response” group showed the highest improvement, followed by the “Diverse and Moderate Responses” group, while the “Steady Response” group exhibited modest or negative shifts.

For “Willingness to Wear” (PreQ6 vs. PostQ4), the “High Anxiety Positive Response” group exhibited the highest positive change, followed by the “Diverse and Moderate Responses” group, while the “Steady Response” group showed the least change.

No cases in this study required escalation for abnormal CTG during the study.

## Discussion

This study, the largest to date exploring pregnant patients’ perceptions of a novel non-invasive fetal ECG (NI-FECG) device for continuous and home fetal monitoring, highlights a strong baseline interest in both applications^5–14^. In the pre-use survey, 90% of respondents expressed interest in home monitoring, 94% perceived continuous fetal monitoring as useful, and 79% were interested in extended monitoring over days or weeks. Nearly 90% anticipated feeling more reassured, and 86% believed it would reduce their anxiety by providing reassurance between scheduled hospital visits. These findings align with previous studies reporting high satisfaction with continuous fetal monitoring, primarily in the intrapartum period. Our study uniquely separates interest in the location of monitoring from interest in continuous monitoring itself, offering insights into patient preferences.

Post-use findings reinforce this high acceptance, with 88.6% reporting overall satisfaction and strong comfort ratings. Our results align with existing literature, such as Crawford et al., who reported a mean satisfaction score of 8.4/10 for the Monica AN24 device, which was preferred for comfort and mobility^9,10,13^. Similarly, Rauf et al. found an average comfort score of 8.3/10 (translated from a four-point Likert score of 3.3 ± 0.6), while our median comfort score was 9 (IQR 2)^12^. Rauf’s work emphasized the benefits of real-time, retrospective monitoring without hospitalization, which enhanced comfort and reduced anxiety compared to CTG^12^. Crawford et al. also highlighted the advantages of continuous monitoring, including non-invasive recording and home-use benefits, which our study supports with a more ergonomic device design^9,10^. Our larger sample allowed for the identification of specific patient subgroups with higher anxiety, enabling targeted support^12^. Furthermore, our post-use satisfaction and comfort scores were significantly higher than those reported in Hamm et al.’s remote fetal monitoring evaluation^5^.

Notably, our findings challenge prior clinician concerns that continuous fetal monitoring might increase maternal anxiety^2,3^. Instead, nearly 90% of participants reported feeling more reassured and less anxious with continuous monitoring. This aligns with systematic reviews demonstrating that remote monitoring can reduce anxiety, improve perinatal outcomes, and lower healthcare costs. Our word cloud analysis further reinforced key themes of decreased anxiety, convenience, and time saved, supporting recent evidence that telehealth and home monitoring are well-received. However, the absence of validated maternal anxiety scores in our study limits direct comparisons with other research.

Cluster analysis of the pre-use survey identified distinct patient attitudes toward home monitoring. Three clusters showed particularly favorable responses, notably among individuals with higher anxiety, BMI, parity, and age, suggesting these subgroups may benefit most from home monitoring interventions. The post-use survey further revealed that the “High Anxiety Group” exhibited significant perception shifts, emphasizing the need for tailored communication strategies to optimize patient engagement. While 76% were comfortable with 24-h monitoring post-use, most participants remained neutral in their preference between NI-FECG and CTG. This could be attributed to familiarity with traditional CTG, particularly the ability to hear the fetal heartbeat, a factor previously identified as influencing patient preference. The concurrent use of CTG alongside NI-FECG in the trial design may also have influenced responses. Additionally, a small subset of participants expressed concerns about prolonged use, citing potential discomfort or interference with daily activities, highlighting the need for further device refinement and patient education. A history of perinatal loss was reported by 40% of participants (majority being first-trimester miscarriage), potentially influencing their interest in continuous monitoring. While clustering analysis did not identify perinatal loss as a defining feature of specific subgroups, its high prevalence suggests it may be an important factor in patient perceptions of monitoring and continuous monitoring should address the concerns and expectations in specific subgroups like these.

This study, one of the largest of its kind, provides comprehensive insights into pregnant patients’ pre-use expectations and post-use experiences with NI-FECG for continuous and home fetal monitoring. Strengths include large sample size, detailed assessments of comfort and satisfaction, and the integration of quantitative surveys with qualitative analysis using natural language processing (NLP) techniques to extract key themes and sentiments^15^. The absence of a validated maternal anxiety score limits comparability with other studies, and recruiting patients already indicated for fetal monitoring may introduce selection bias. Additionally, factors influencing reluctance to participate were not fully explored. Future research should validate the acceptability and efficacy of remote fetal monitoring across diverse populations while addressing practical concerns such as device fit and long-term comfort.

Our study complements and advances other contemporary research and innovation initiatives by incorporating real-world pre-use and post-use experiences with a novel NI-FECG device. Wakefield et al. conducted a large online survey, finding that over 90% of women were willing to use a wearable fetal ECG device, but their study assessed hypothetical interest^16^. In contrast, our study captures actual user experience, evaluating comfort, feasibility, and satisfaction after device usage. While Wakefield et al. relied on structured surveys, we applied NLP-driven sentiment analysis and clustering techniques to uncover subgroup-specific insights in addition to structured questions. Recent initiatives like PowerMom, a large-scale maternal health research program by Scripps Research, use wearable technology to gather broad population-level data on maternal-fetal health^17^. While PowerMom provides valuable big-data insights, our study complements rather than competes by offering granular, patient-centered perspectives in a clinical setting. By bridging the gap between large-scale data collection and individual user experience insights, our findings further support the integration of wearable fetal monitoring technologies into clinical practice.

Although this is the largest study on pregnant patients’ perceptions of NI-FECG, a larger sample is needed for widespread deployment. Scaling digital maternal health research requires a structured, multi-phase approach. Future studies should begin with a multi-center validation involving several hundred participants to assess inter-site variability, usability, and reproducibility. This should be followed by a larger trial integrating real-world home monitoring to evaluate adherence, long-term acceptability, and clinical outcomes, with COMFY1, 2, and 3 trials from our research group contributing to these foundational phases. Future trials (including pivotal trials for regulatory approval) will assess comparative effectiveness, cost-benefit, and clinician workflow integration.

Our study advances prior work, including Wakefield et al., by aligning with regulatory and funding priorities that emphasize pre- and post-use patient experience^16^. Unlike purely cross-sectional surveys, our methodology captures sustained engagement, patient adherence, and real-world feasibility, providing critical insights for clinical translation. Informed by consumer feedback, we have incorporated user-driven refinements into subsequent iterations of the commercial-grade NI-FECG device, which can be accessed via the Kali Healthcare website^18^. Furthermore, our separate sub-study has reported on the performance of the NI-FECG device compared to conventional CTG and optimized an artificial intelligence algorithm to extract the fetal heart rate. In a validation cohort of 70 pregnant women, the AI algorithm demonstrated 90.4% reliability (95% CI: 87.0–92.7%) in detecting fetal heart rate compared to CTG, with no signal dropout throughout the monitoring period^19^.

In conclusion, NI-FECG shows strong potential for home and continuous fetal monitoring, with high comfort and reassurance levels among participants. However, the neutral preference between NI-FECG and CTG suggests further refinement in device design, education, and clinical protocols is needed. Future research should explore the impact of extended home monitoring on anxiety and address practical concerns to enhance long-term acceptability.

## Methods

This perception study was conducted as part of the umbrella COMFY2 (Continuous fetal heart rate Monitoring using non-invasive Fetal electrocardiographY) trial, which evaluated the reliability of a novel non-invasive fetal ECG (NI-FECG) device (Fig. 1) compared to standard cardiotocography (CTG) monitoring. The COMFY2 study is a nonrandomized feasibility study.

### Participants and Study Design

Eligible participants were recruited by a research midwife or doctor if scheduled for routine fetal monitoring from 36 weeks’ gestation with a singleton pregnancy. Exclusion criteria included age under 18, over 45, and non-English speakers. Of the 71 patients approached, 70 completed pre- and post-use questionnaires; one withdrew. The sample size was based on the primary outcome of the COMFY2 study, consistent with similar research on continuous fetal monitoring. Recruitment occurred consecutively between November 2021 and April 2022 during antepartum visits at the fetal monitoring unit of Mercy Hospital for Women, a tertiary hospital in Melbourne, Australia. This unit serves as an outpatient high-risk pregnancy assessment center. All participants had a risk-related indication for routine antepartum fetal monitoring and were recruited during scheduled visits. No preselection based on demographic or clinical factors ensured a representative antenatal sample (Table 1).

### Data collection

Participants wore the NI-FECG device for 30 min alongside conventional cardiotocography (CTG) monitoring (ultrasound mode) to ensure clinical safety while assessing real-world user experience.

Structured, face-validated pre- and post-use questionnaires (Fig. 2 and supplementary information) were used to evaluate perceptions of the NI-FECG device. The pre-use questionnaire assessed interest, perceived usefulness, comfort, and anxiety related to continuous and home monitoring, including willingness to use the device for extended periods (days to weeks) and at home. Responses were rated on a Likert scale (1–10), with free-text fields for justification. The post-use questionnaire collected feedback on actual device experience, including comfort, ease of removal, skin irritation, satisfaction, and willingness for extended use. Participants also provided qualitative feedback on desired features for future iterations.

To enhance qualitative insights, Likert scale responses were supplemented with NLP-based sentiment analysis. Surveys were completed before and after device use in the fetal monitoring unit with the research midwife.

For this study, “continuous” fetal monitoring refers to extended monitoring beyond handheld intermittent Doppler use, aligning with the standard definition of continuous electronic fetal monitoring (CEFM, that typically would be 20 min or more). This includes prolonged use (≥24 h) and at-home monitoring^20^. Participants wore the NI-FECG patch for 30 min while being surveyed on their perceptions of longer-term use, aligning with prior studies that set 30 min as the minimum duration^21^.

### Validation of questionnaires

To assess the construct and discriminant validity of the questionnaires, rank correlation analyses were performed on participant responses for both pre-use and post-use surveys. Positive correlations were observed between Q1 (usefulness of continuous fetal monitoring), Q2 (interest in wearing a monitor), and Q3 (reassurance from monitoring), supporting construct validity. A negative correlation was found between Q4 (anxiety towards continuous monitoring) and Q1–Q3, indicating discriminant validity. Additionally, high correlations between Q5 (willingness to wear the device for 24 h) and Q6 (satisfaction with the device) suggest these questions measure similar constructs. For the post-use questionnaire, high correlations were identified between Q1 (comfort with wearing the device) and Q4 (willingness for extended wear), Q4 and Q6 (overall satisfaction), and Q1 and Q6, all exceeding 0.4. These correlations indicate that comfort and willingness for extended wear significantly influence overall satisfaction. Conversely, Q3 (skin irritation) demonstrated negative correlations with other questions, highlighting its adverse effect on satisfaction and comfort. The remaining items showed moderate correlations, providing further evidence of the questionnaire’s validity.

### Data analysis

Statistical analysis was conducted using R (4.3.1), Jamovi (2.4.8.0), and Python (3.10). Quantitative data were visualized and tested for skewness, and results were presented as means (SD), medians (IQR), or proportions. The pre- and post-use responses were compared using the Wilcoxon signed-rank test to analyze changes over time, and McNemar’s test was used to assess the binarized high (≥7) versus low scores. Due to the nonparametric distribution and multicollinearity, hierarchical cluster analysis using Scipy and Pandas with Hamming Distance and k-means was employed to identify patient groups for personalized clinical strategies.

### Natural language processing and matching

NLP libraries (Word Cloud 1.8.1, TextBlob 0.17.1) were used for sentiment analysis of free-text responses, identifying themes such as reassurance, convenience, and anxiety reduction^15^. An NLP semantic matching library was employed to align pre- and post-use questions with common themes to assess perceived utility versus actual satisfaction, initial interest versus comfort, and willingness to use the device for varying durations. This approach was intended to allow for the identification of participant clusters, aiding in the development of targeted education and marketing strategies.

### Clinical context

This study focused on user perceptions and experiences rather than comparing NI-FECG with CTG for diagnostic performance. NI-FECG was used alongside standard CTG (ultrasound mode), which remained the primary monitoring tool. As per the study protocol, informed consent specified that any abnormal CTG findings (with NI-FECG used concurrently) would be managed per hospital protocol, with no clinical decisions based on NI-FECG. The diagnostic accuracy of NI-FECG is reported separately as a sub-study.

### Details of ethics approval

The study protocol was prospectively approved by the Mercy Public Hospitals (Victoria) institutional Human Research Ethics Committee in August 2021 (HREC 2021-043). The trial is prospectively registered on Australia New Zealand Clinical Trials Registry which is a primary registry in the WHO (World Health Organisation) registry network (ANZCTRN12621001260819; submitted June 9th, 2021; approved September 17th, 2021). Informed consent for study participation was obtained from all participants.

## Supplementary information

supplementary information

## Data Availability

All details of trial protocols and individual-level data of the included cohort can be shared upon reasonable request to the corresponding author and completion of data transfer agreement forms. The NI-FECG device is proprietary and patented.

## Code availability

The Python code, and data key for variables, for data preprocessing, statistical analysis, and NLP/cluster modeling is available upon reasonable request from the corresponding author, subject to completion of data transfer agreement forms.
