# Supplementary material for: Consumer insights from a feasibility study on remote and extended use of a novel non-invasive wearable fetal electrocardiogram monitor
Source: NPJ Digit Med. 2025 Apr 21;8:216. doi: 10.1038/s41746-025-01628-9 (PMC12012179; doi:10.1038/s41746-025-01628-9)
Supplement: Supplementary file 1 — supplementary information [file 41746_2025_1628_MOESM1_ESM.pdf]

CONSORT 2010 checklist of information to include when reporting a pilot or feasibility trial*

Section/Topic	Item No	Checklist item	Reported on page No
**Title and abstract**			
	1a	Identification as a pilot or feasibility randomised trial in the title	Page 1
	1b	Structured summary of pilot trial design, methods, results, and conclusions (for specific guidance see CONSORT abstract extension for pilot trials)	Page 2
**Introduction**			
Background and objectives	2a	Scientific background and explanation of rationale for future definitive trial, and reasons for randomised pilot trial	Pages 3,10
	2b	Specific objectives or research questions for pilot trial	Pages 3,4,14
**Methods**			
Trial design	3a	Description of pilot trial design (such as parallel, factorial) including allocation ratio	Page 14,15
	3b	Important changes to methods after pilot trial commencement (such as eligibility criteria), with reasons	Not applicable – no changes reported
Participants	4a	Eligibility criteria for participants	Page 14
	4b	Settings and locations where the data were collected	Page 14
	4c	How participants were identified and consented	Page 14,15
Interventions	5	The interventions for each group with sufficient details to allow replication, including how and when they were actually administered	Page 15,16
Outcomes	6a	Completely defined prespecified assessments or measurements to address each pilot trial objective specified in 2b, including how and when they were assessed	Page 15-17
	6b	Any changes to pilot trial assessments or measurements after the pilot trial commenced, with reasons	Not applicable – no changes reported
	6c	If applicable, prespecified criteria used to judge whether, or how, to proceed with future definitive trial	Page 13
Sample size	7a	Rationale for numbers in the pilot trial	Page 14
	7b	When applicable, explanation of any interim analyses and stopping guidelines	Not applicable – no interim analysis or

			stopping rule
Randomisation:			
Sequence generation	8a	Method used to generate the random allocation sequence	Not applicable – this was a non-randomised pilot study
	8b	Type of randomisation(s); details of any restriction (such as blocking and block size)	Not applicable – this was a non-randomised pilot study
Allocation concealment mechanism	9	Mechanism used to implement the random allocation sequence (such as sequentially numbered containers), describing any steps taken to conceal the sequence until interventions were assigned	Not applicable – this was a non-randomised pilot study
Implementation	10	Who generated the random allocation sequence, who enrolled participants, and who assigned participants to interventions	Not applicable – this was a non-randomised pilot study
Blinding	11a	If done, who was blinded after assignment to interventions (for example, participants, care providers, those assessing outcomes) and how	Not applicable – no blinding; all participants knew they were trial participants. Participants were approached sequentially

			with no preselection (page 16)
	11b	If relevant, description of the similarity of interventions	Not applicable – all received NI-FECG + standard CTG
Statistical methods	12	Methods used to address each pilot trial objective whether qualitative or quantitative	Page 15-17
**Results**			
Participant flow (a diagram is strongly recommended)	13a	For each group, the numbers of participants who were approached and/or assessed for eligibility, randomly assigned, received intended treatment, and were assessed for each objective	Page 4,14
	13b	For each group, losses and exclusions after randomisation, together with reasons	Page 14
Recruitment	14a	Dates defining the periods of recruitment and follow-up	Page 14
	14b	Why the pilot trial ended or was stopped	Trial completed as planned
Baseline data	15	A table showing baseline demographic and clinical characteristics for each group	Table 1
Numbers analysed	16	For each objective, number of participants (denominator) included in each analysis. If relevant, these numbers should be by randomised group	Page 4, Results – 70 completed; analysis includes all completers
Outcomes and estimation	17	For each objective, results including expressions of uncertainty (such as 95% confidence interval) for any estimates. If relevant, these results should be by randomised group	Pages 4-9; tables1,2;supplementary information
Ancillary analyses	18	Results of any other analyses performed that could be used to inform the future definitive trial	Pages 4-9
Harms	19	All important harms or unintended effects in each group (for specific guidance see CONSORT for harms)	Results – Minimal skin irritation (2.9%), no Pages 7,9

			escalation for abnormal CTG
	19a	If relevant, other important unintended consequences	Page 11,13
**Discussion**			
Limitations	20	Pilot trial limitations, addressing sources of potential bias and remaining uncertainty about feasibility	Page 12
Generalisability	21	Generalisability (applicability) of pilot trial methods and findings to future definitive trial and other studies	Page 13
Interpretation	22	Interpretation consistent with pilot trial objectives and findings, balancing potential benefits and harms, and considering other relevant evidence	Pages 12- 14
	22a	Implications for progression from pilot to future definitive trial, including any proposed amendments	Pages 13,14
**Other information**			
Registration	23	Registration number for pilot trial and name of trial registry	Page 18
Protocol	24	Where the pilot trial protocol can be accessed, if available	Page 18
Funding	25	Sources of funding and other support (such as supply of drugs), role of funders	Page 18
	26	Ethical approval or approval by research review committee, confirmed with reference number	Page 17

Citation: Eldridge SM, Chan CL, Campbell MJ, Bond CM, Hopewell S, Thabane L, et al. CONSORT 2010 statement: extension to randomised pilot and feasibility trials. BMJ. 2016;355. This is an Open Access article distributed in accordance with the terms of the Creative Commons Attribution (CC BY 3.0) license (http://creativecommons.org/licenses/by/3.0/), which permits others to distribute, remix, adapt and build upon this work, for commercial use, provided the original work is properly cited.

*We strongly recommend reading this statement in conjunction with the CONSORT 2010, extension to randomised pilot and feasibility trials, Explanation and Elaboration for important clarifications on all the items. If relevant, we also recommend reading CONSORT extensions for cluster randomised trials, non-inferiority and equivalence trials, non-pharmacological treatments, herbal interventions, and pragmatic trials. Additional extensions are forthcoming: for those and for up-to-date references relevant to this checklist, see www.consort-statement.org.

Questionnaire before use of the of the baby heartbeat monitor device

Q1) Do you think a device that continuously monitors your baby's heartbeat in pregnancy over days or weeks could be useful?

1	2	3	4	5	6	7	8	9	10
Not useful									Very useful

Q2) How interested are you to wear a monitor that continuously records your baby's heartbeat over days or weeks?

1	2	3	4	5	6	7	8	9	10
Not interested				Neutral					Very interested

Q3) Do you think a monitor that continuously records your baby's heartbeat over days or weeks would make you feel more or less reassured?

1	2	3	4	5	6	7	8	9	10
Less reassured				Neutral					More reassured

Q3a) Please explain your answer to this.

Q4) Do you think a monitor that continuously records your baby's heartbeat over days or weeks would make you feel more or less anxious?

1	2	3	4	5	6	7	8	9	10
Less anxious				Neutral					More anxious

Q4a) Please tell us why?

Q5) Would you be happy to wear a baby heartbeat monitor device for 24 hours?

1	2	3	4	5	6	7	8	9	10
Not happy to wear									Very happy to wear

Q5a) Please tell us why?

Q6) Would you be happy to wear a fetal heartbeat monitor device for longer than 24 hours over days or weeks?

1	2	3	4	5	6	7	8	9	10
Not happy to wear									Very happy to wear

Q6a) Please tell us why?

Q7) Would you be happy to wear this device at home?

1	2	3	4	5	6	7	8	9	10

Prefer to wear at hospital only — Very happy to wear at home

Q6a) Please tell us why?

Q8) If you have a complicated pregnancy or if your pregnancy became complicated would you be more or less likely to want to wear a fetal monitoring device?

1	2	3	4	5	6	7	8	9	10

I wouldn’t want to wear the device — I would want to wear the device

Q8a) If you have a complicated pregnancy, please tell us why?

Q9) What features would you want a continuous baby heart rate monitor to have? You can describe or draw this below.

Questionnaire following use of the of the baby heartbeat monitor device

Q1) How comfortable was the continuous baby heartbeat device to wear?

1	2	3	4	5	6	7	8	9	10
Uncomfortable									Comfortable

Q1a) Please tell us why?

___

Q2) How comfortable was it to take the sensor patch off?

1	2	3	4	5	6	7	8	9	10
Uncomfortable									Comfortable

Q2a) If the sensor patch was uncomfortable to take off, please explain why?

___

Q3) Was your skin irritated (i.e. rash or marks on the skin)?

1	2	3	4	5	6	7	8	9	10
No irritation									Lots of irritation

Q3a) Please describe your skin irritation if it did happen it and how long it lasted?

___

Q4) Would you be happy to wear a device like this for 24 hours or longer?

1	2	3	4	5	6	7	8	9	10
Not happy to wear									Very happy to wear

Q4a) Please tell us why?

___

Q5) Do you prefer the new fetal heartbeat device compared to the existing CTG device?

1	2	3	4	5	6	7	8	9	10

Prefer the CTG device — No preference — Prefer the new device

Q5a) Please tell us why?

____

Q6) Overall how satisfied were you with the new baby heartbeat device?

1	2	3	4	5	6	7	8	9	10

Not at all satisfied — Very satisfied

Q6a) Please explain your answer to this?

____

Q7) What features would you want a continuous baby heart rate monitor to have? You can describe or draw this below.

____

____

____

____

Thank you for completing this questionnaire

SUPPLEMENTARY TABLE 1: OCCUPATION AND COUNTRY OF BIRTH OF PARTICIPANTS

Characteristic	N=70
**ANZSCO occupation code, Count (Percentage)**	
Home Duties	7(10%)
Not available/Not stated	9(12.86%)
Registered Nurses	2(2.86%)
Nursing Support and Personal Care Workers	2(2.86%)
Retail Managers	2(2.86%)
Early Childhood (Pre-primary School) Teachers	3(4.29%)
Other Engineering Professionals	1(1.43%)
Advertising and Marketing Professionals	1(1.43%)
Other Miscellaneous Clerical and Administrative Workers	12(17.14%)
Specialist Physicians	2(2.86%)
Environmental Scientists	1(1.43%)
Other Specialist Managers	1(1.43%)
Public Servant	2(2.86%)
Hairdressers	2(2.86%)
Office Managers	2(2.86%)
Medical Imaging Professionals	1(1.43%)
Welfare Support Workers	2(2.86%)
Secondary School Teachers	1(1.43%)
Audiologists and Speech Pathologists / Therapists	1(1.43%)
Clothing Trades Workers	1(1.43%)
Child Carers	1(1.43%)
Dental Assistants	1(1.43%)
Recruitment Consultant	1(1.43%)
Occupational Therapist	1(1.43%)
Human Resource Professionals	2(2.86%)

Nursing Support and Personal Care Workers	1(1.43%)
Architects and Landscape Architects	2(2.86%)
Occupational and Environmental Health Professionals	1(1.43%)
Florists	1(1.43%)
Psychologists	1(1.43%)
Tourism and Travel Advisers	1(1.43%)
Primary School Teachers	1(1.43%)
Real Estate Sales Agents	1(1.43%)
**Country of Birth SACC 2016 code, Count(Percentage)**	
Oceania and Antarctica	51 (73%)
North-West Europe	3 (4.3%)
Southern and Eastern Europe	1 (1.4%)
North Africa and Middle East	2 (2.9%)
South-East Asia	1 (1.4%)
North-East Asia	1 (1.4%)
Southern and Central Asia	4 (5.7%)
Americas	2 (2.9%)
Sub-Saharan Africa	5 (7.1%)

SUPPLEMENTARY TABLE 2: NLP STATISTICS ON OPEN ENDED RESPONSES FOR PRE-USE QUESTIONNAIRE

Question	Mean	SD	Min	Max
Q3: Do you think a monitor that continuously records your baby's heartbeat over days or weeks would make you feel more or less reassured?	0.18	0.26	-0.5	0.7
Q4: Do you think a monitor that continuously records your baby's heartbeat over days or weeks would make you feel more or less anxious?	0.08	0.28	-0.5	0.67
Q5: Would you be happy to wear a baby heartbeat monitor device for 24 hours?	0.10	0.34	-0.8	1.0
Q6: Would you be happy to wear a fetal heartbeat monitor device for longer than 24 hours over days or weeks?	0.04	0.34	-0.8	0.8
Q7: Would you be happy to wear this device at home?	0.23	0.26	-0.29	0.8
Q8: Would you be happy to wear this device at home?	0.21	0.27	-0.5	0.8
Q9: What features would you want a continuous baby heart rate monitor to have? You can describe or draw this below.	0.13	0.27	-0.25	1.0

NB: NLP=Natural Language Processing

SUPPLEMENTARY TABLE 3: KEY FEATURES OF CLUSTERS BEFORE USE OF DEVICE

Cluster	Trial ID	Distribution of rating to Q4[a] [% high ratings; Median (IQR)]	Sentiment analysis Q4 text responses	Distribution of rating to Q7[b] [% high ratings; Median (IQR)]	Sentiment analysis Q7 text responses	Anxiety/Depression	Maternal Age	Gestation	Country of birth (Top 3)	Occupation (Top 3)	Parity	Booking BMI	Indication for monitoring
1(Neutral Expectation Group)	15, 6, 52, 40, 11, 7, 48, 70, 74, 1, 25, 27, 21	7.7%; 5(3) Display a broad range of ratings with the median lying around the middle representing variety of opinions.	Most responses seem to have a neutral sentiment, with a few leaning slightly negative or positive. Broadest interquartile range, indicating varied sentiments	53.8%;8(5) Particularly wide range of responses, while maintaining a high median rating	Responses mostly neutral with a slightly positive skew. The sentiment is less varied than in Q4.	Has a moderate number of individuals without anxiety, significant number of individuals without depression	Most individuals in their late 20s to early 30s	Most individuals between 38 and 39 weeks.	Significant number of individuals from Oceania-Antarctica region	Demonstrates diversity in occupations.	Follows behind Cluster 2 in average parity.	The distribution suggests a wide range of BMI	Varied distribution of indications for monitoring.
2(Cautiously Interested Diverse Group)	8, 71, 73, 23, 65, 13, 34, 3, 28, 35, 61, 68, 20, 43, 69, 18, 46, 2, 24, 42, 64, 49, 39, 59, 47, 5, 19, 26, 58	10.3%;2(4) Display a broad range of ratings with the median lying around the middle of the scale, suggesting a variety of opinions on Q4.	Notable peak in neutral sentiment, with a few responses on either side of the sentiment spectrum.	96.6%;9(2) Suggests generally positive opinion of Q7	The sentiment shows a clear positive peak, implying that responses are generally positive about Q7	Highest number of individuals without anxiety and depression.	This cluster is showing a particularly high count in the early 30s range.	Most individuals between 38 and 39 weeks.	Displays a very high count for Oceania-Antarctica region	There is a notable peak for "Home Duties".	Has the highest average parity	Noticeable peak in BMI for Cluster 2	Prominent peak for Hypertension in pregnancy
3(Optimistic Group)	63, 54, 51, 17, 62, 29, 67, 37, 22, 50, 41, 38, 12, 30, 44, 45, 4	35.3%;3(6) Presents the highest median rating	The sentiment is overwhelmingly positive with most responses falling in the positive range. This cluster has the highest median sentiment.	100%;10(1) Suggests generally positive opinion of Q7	Dominant dominant positive sentiment.	Relatively balanced counts between those with and without anxiety, with. More individuals not reporting depression than those reporting it.	Most individuals are in their late 20s to early 30s.	Most individuals between 38 and 39 weeks.	Evenly distributed among its top three countries.	"Clerical and Administrative Workers" is dominant	Has a notably lower average parity.	The distribution suggests a wide range of BMI	High count of "Other/Miscellaneous" indication of monitoring.
4(Mixed Positive Group)	57, 72, 14, 16, 10, 9, 66, 36, 56, 60, 75	NA;2(0.5)[c] Shows a lower median rating.	Similar to Cluster 1, balanced distribution with a skew towards neutral-positive sentiment.	100%;10(1) Suggests generally positive opinion of Q7	The positive sentiment is predominant, but there are a few neutral or less positive responses, indicating a slight variation in opinions.	Relatively balanced counts between those with and without anxiety/depression.	Most individuals across clusters are in their late 20s to early 30s.	Most individuals between 38 and 39 weeks.	Shows diversity in the top three countries of birth.	Shows an even distribution among the top three occupations.	Has the lowest average parity.	The distribution suggests a wide range of BMI	No Post term or GDM in this cluster

[a]Q4: Do you think a monitor that continuously records your baby's heartbeat over days or weeks would make you feel more or less anxious;[b]Q7: Would you be happy to wear this device at home?;[c]IQR values calculated via interpolation may result in non-integer values despite integer-only responses in the dataset.

Supplementary Table 4: Sentiment Analysis Results after use of device

Open Ended Question	Stem Question	Polarity	Subjectivity
Q1a) Please tell us why?	Q1) Comfort of Wearing the Device	0.031	0.411
Q2a) If the sensor patch was uncomfortable to take off, explain why?	Q2) Comfort of Removing the Sensor	-0.014	0.43
Q3a) Please describe your skin irritation if it happened and how long it lasted?	Q3) Skin Irritation	0.011	0.256
Q4a) Please tell us why?	Q4) Willingness to Wear Device for 24h+	0.154	0.521
Q5a) Please tell us why?	Q5) Preference for New Device Over CTG	0.119	0.326
Q7) What features would you want a continuous baby heart rate monitor to have?		0.185	0.423

SUPPLEMENTARY TABLE 5: CHANGE IN SCORES OF ALIGNED QUESTION PAIRS FROM PRE-USE TO POST-USE FOR WHOLE COHORT

Question Pair	Wilcoxon Signed-Rank Test Statistic on actual ratings[a]	p-Value Wilcoxon Signed-Rank	Pre-use Median rating	Post-use Median rating	Change Direction	McNemar test Statistic on binarized rating	p-Value McNemar test	Change Direction
PreQ1 vs. PostQ6 (Usefulness vs. Satisfaction)	169.5	<0.0001	10	9	**Decreased**	0.75	0.39	No Change
PreQ2 vs. PostQ1 (Interest vs. Comfort)	457.5	0.08	9	9	No Change	3.06	0.08	No Change
PreQ6 vs. PostQ4 (Willingness to Wear Short vs. Long Term)	162.0	<0.0001	6	8	**Increased**	16.00	<0.0001	**Increased**

[a] data was non parametrically distributed

SUPPLEMENTARY TABLE 6: KEY FEATURES OF CLUSTERS AFTER USE OF DEVICE

Feature	Cluster 0 *"High Anxiety Positive Response Group"*	Cluster 1 *"Steady Response group"*	Cluster 2 *"Diverse and Moderate Responses Group*	Comments
Cluster size	11	11	48	
Maternal Age	32.5 years	34.0 years	31.96 years	
Parity-median,(IQR)	0(1)	1(1.5)[a]	1(1)	
Anxiety counts	10(high)	1(Lowest)	4	
Depression counts	8(High)	0	0	
Number of participants by indications of monitoring	Decreased Fetal Movements: 2<br>Hypertension in Pregnancy: 6<br>Intra-Uterine Growth Restriction (IUGR): 1<br>Gestational Diabetes: 0<br>Post Term/Dates: 0<br>Other: 2	Decreased Fetal Movements: 4<br>Hypertension in Pregnancy: 3<br>Intra-Uterine Growth Restriction(IUGR): 2<br>Gestational Diabetes: 2<br>Post Term/Dates: 0<br>Other: 0	Decreased Fetal Movements: 10<br>Hypertension in Pregnancy: 14<br>Intra-Uterine Growth Restriction(IUGR): 10<br>Gestational Diabetes: 3<br>Post Term/Dates: 4<br>Other: 7	
PostQ Scores	High counts for binarized high scores	Moderate to low counts for binarized	Generally high scores except for Q3(2 counts)	

	Q1(11), Q2(10), Q4(11) and Q6(10), indicating positive responses.	high scores, notably low for Q4(0 counts).	and Q5(14 counts).<br><br>Counts for high binarized scores:<br>Q1:46,<br>Q1:48,<br>Q1: 42,<br>Q1:45	
Change of score in aligned pairs	High change of score in aligned pairs indicating significant changes from pre to post.	Lower change of score in aligned pairs, suggesting smaller changes from pre to post.	Moderate change of score in aligned pairs	
Question pair	Median	Median	Median	Mann Whitney U/Kruskal Wallis Test Statistic
ChangeQ1preQ6post	9.0(0)	8.0(1.5)	9.0(0)	5.53, P= 0.01
ChangeQ2preQ1post	9.0(1)	4.0(3.5)	8.0(2)	13.91, P<0.01
ChangeQ6preQ4post	8.0(2.0)	3.0(4.0)	5.0(3.0)	8.65, P<0.01

[a] *Mean higher than other clusters ,1.64,SD 1.96*

SUPPLEMENTARY TABLE 7: TYPE OF SENTIMENT CHANGE FROM Q6 PRE TEXT TO Q4 POST TEXT(WILLINGNESS TO WEAR THE DEVICE SHORT VERSUS LONG TERM DOMAIN), STRATIFIED BY CLUSTER

**Change Type**	**Count (Proportion %)**	**95% CI Lower Upper**	**Count (Proportion %)**	**95% CI Lower Upper**	**Count (Proportion %)**	**95% CI Lower Upper**
	**Cluster 0*“High Anxiety Positive Response Group”***		**Cluster 1*“Steady Response group”***		**Cluster 2*“Diverse and Moderate Responses Group”***	
No Data	4 (36.36%)	7.94% 64.79%	5 (45.45%)	16.03% 74.88%	17 (35.42%)	21.89% 48.95%
No Change	4 (36.36%)	7.94% 64.79%	2 (18.18%)	-4.61% 40.97%	13 (27.08%)	14.51% 39.65%
Neutral to Positive	1 (9.09%)	-7.90% 26.08%	1 (9.09%)	-7.90% 26.08%	7 (14.58%)	4.60% 24.57%
Neutral to Negative	1 (9.09%)	-7.90% 26.08%	1 (9.09%)	-7.90% 26.08%	4 (8.33%)	0.51% 16.15%
Negative to Neutral	1 (9.09%)	-7.90% 26.08%	1 (9.09%)	-7.90% 26.08%	4 (8.33%)	0.51% 16.15%

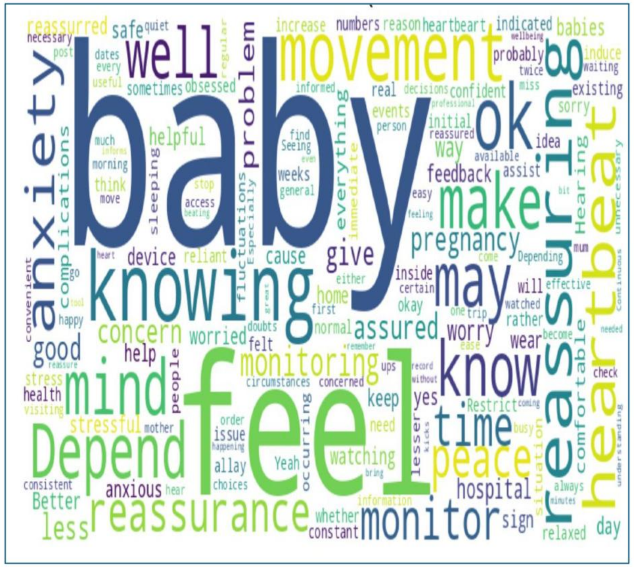

a

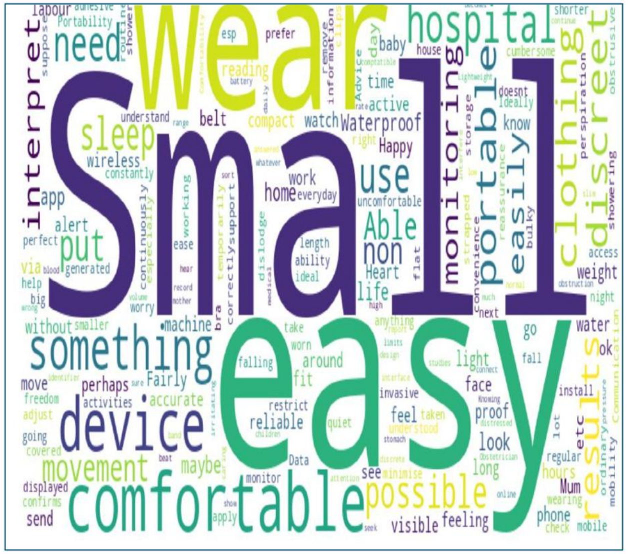

b

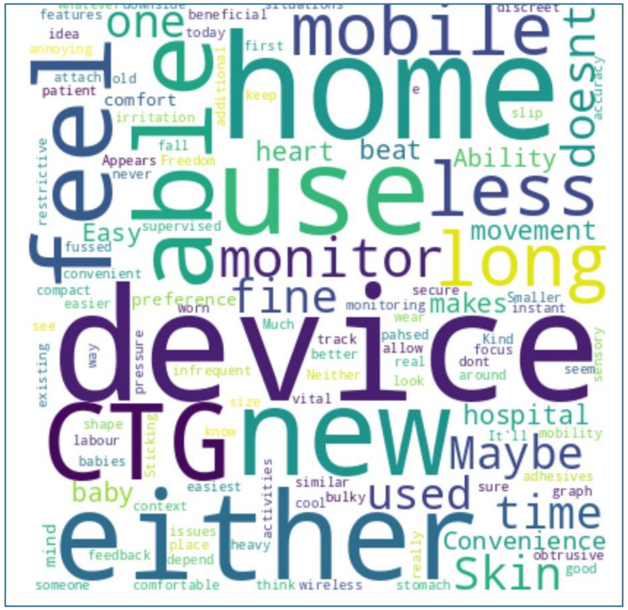

c

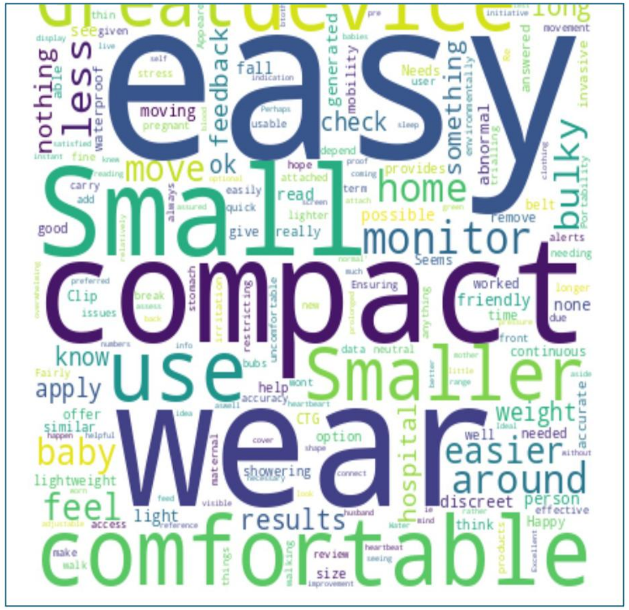

d

Supplementary figure 1

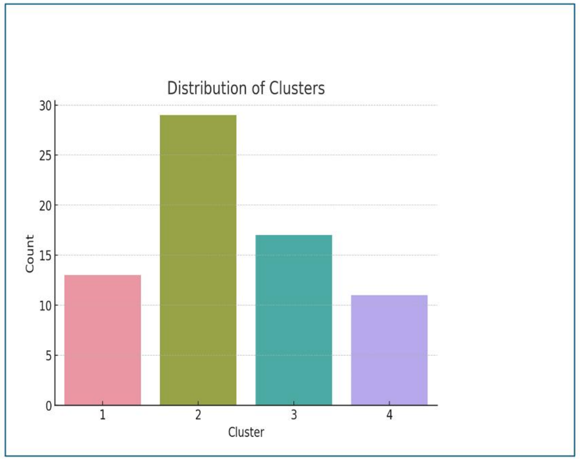

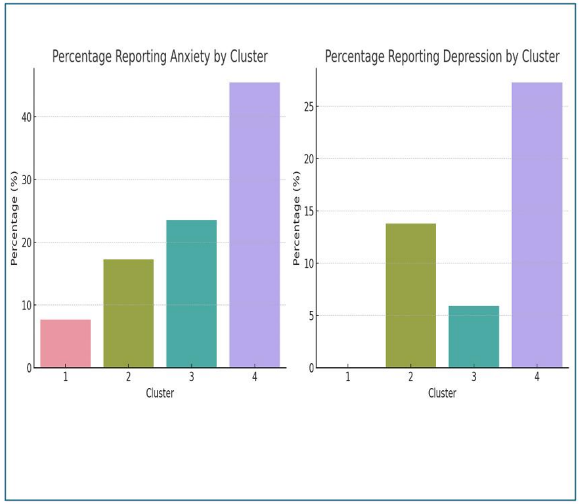

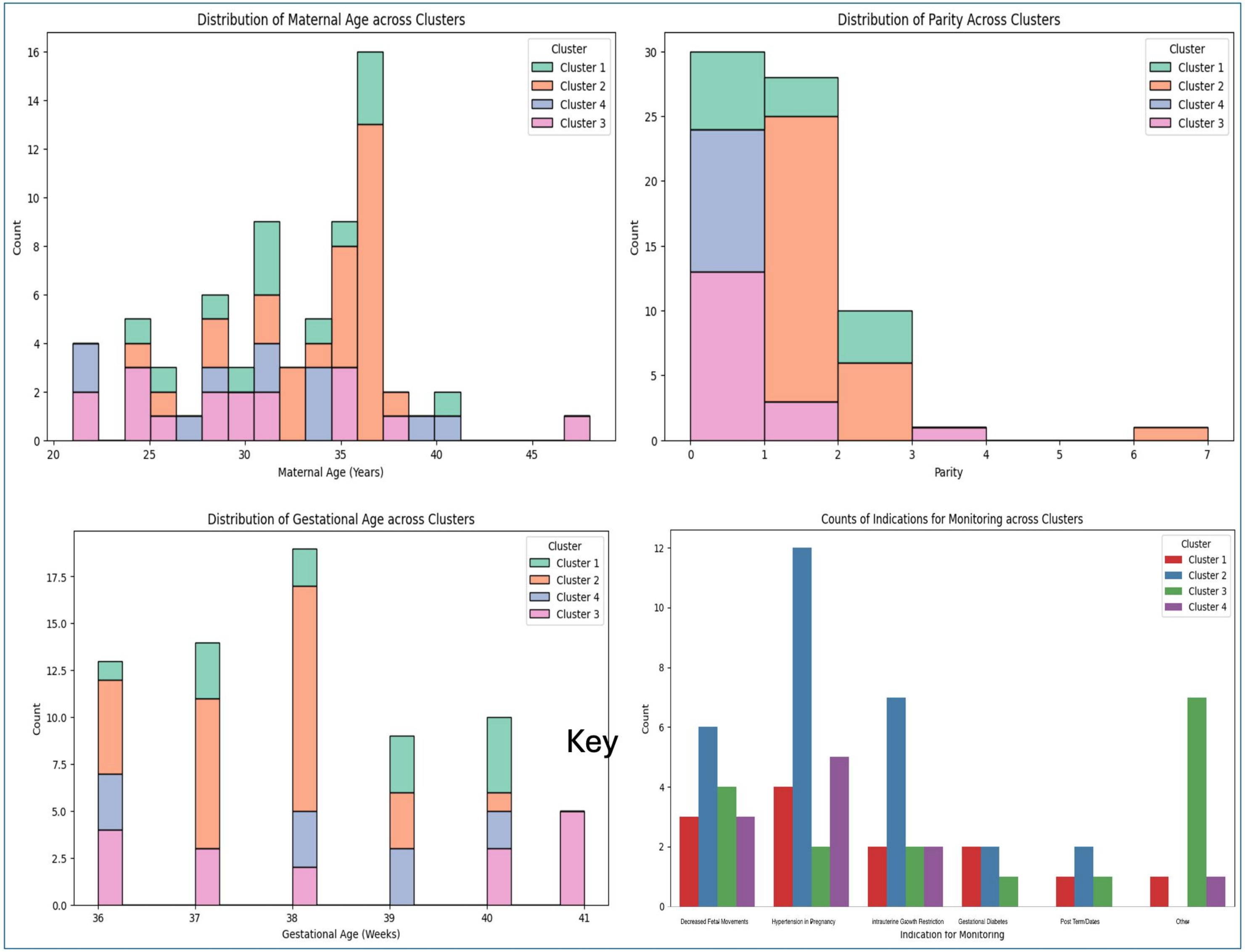

<u>Key for clusters based on pre-use responses</u>

1. Neutral Expectation Group
2. Cautiously Interested Diverse Group
3. Optimistic Group
4. Mixed Positive Group

Supplementary figure 2

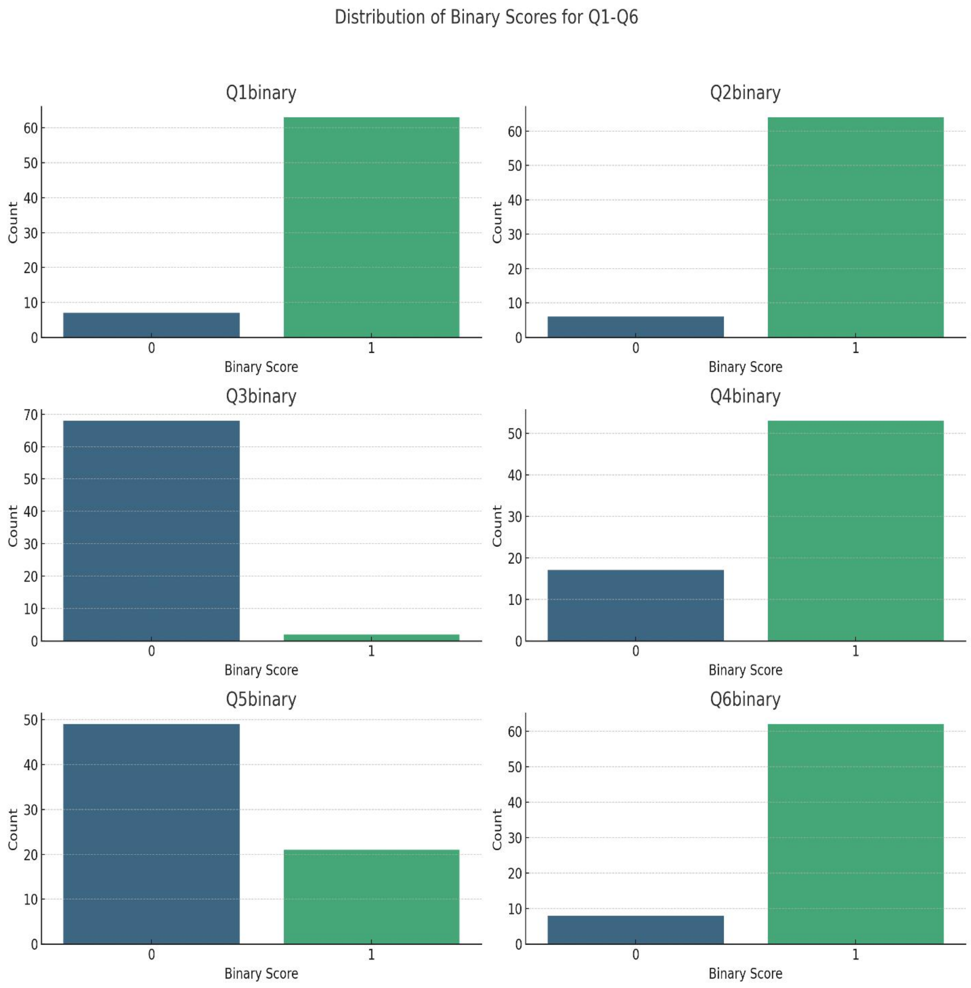

Post-use Questionnaire

Q1) How comfortable was the continuous baby heartbeat device to wear?

Q2) How comfortable was it to take the sensor patch off?

Q3) Was your skin irritated (i.e. rash or marks on the skin)?

Q4) Would you be happy to wear a device like this for 24 hours or longer?

Q5) Do you prefer the new fetal heartbeat device compared to the existing CTG device?

Q6) Overall how satisfied were you with the new baby heartbeat device?

Supplementary figure 3

Q4“Do you think a monitor that continuously records your baby’s heartbeat over days or weeks would make you feel more or less anxious? Please tell us why”

Q7“Would you be happy to wear this device at home? Please tell us why ?”

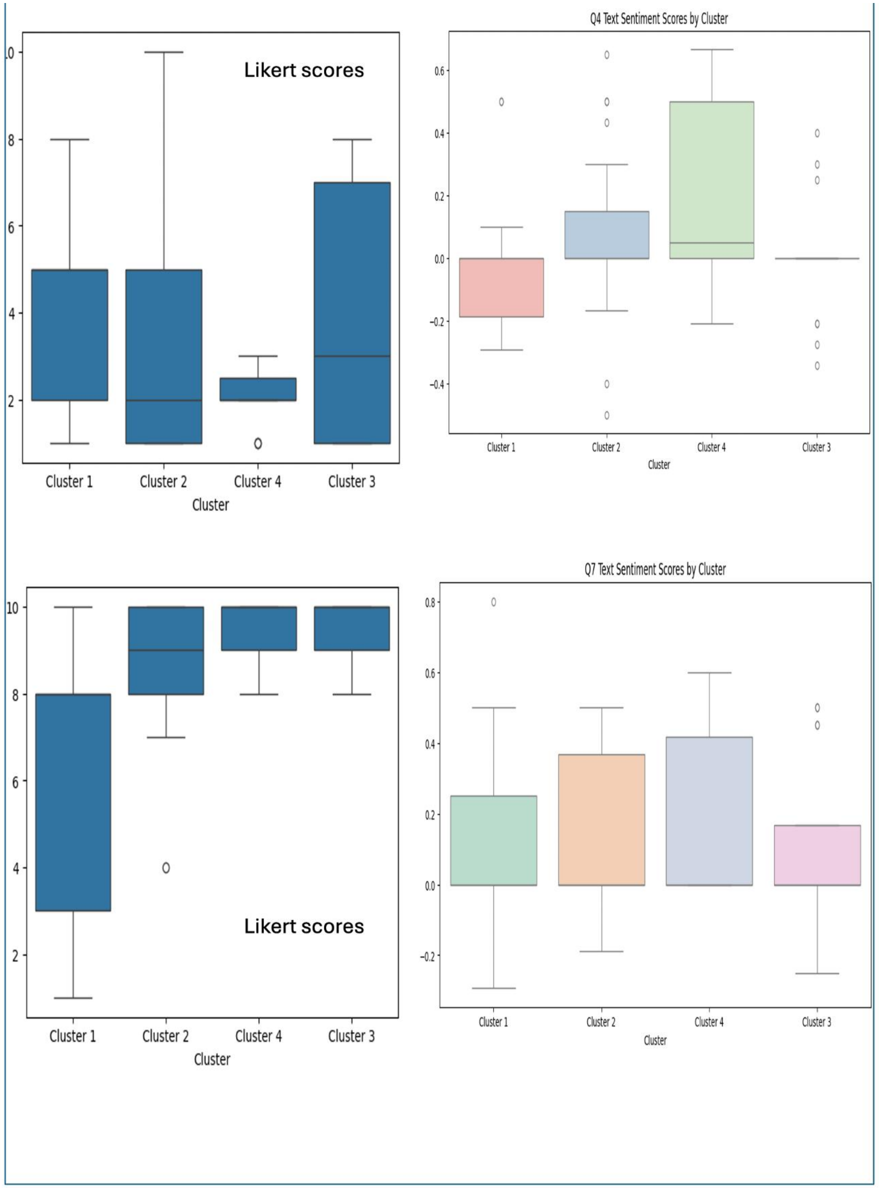

Key for clusters based on pre-use responses

1. Neutral Expectation Group
2. Cautiously Interested Diverse Group
3. Optimistic Group
4. Mixed Positive Group

Supplementary figure 4

Legend for Supplementary tables

Supplementary table 1: Occupation and country of birth of participants

Supplementary table 2: NLP statistics on open-ended responses for pre-use questionnaire

Supplementary table 3: Key features of clusters before use of device

Supplementary table 4: Sentiment analysis results after use of device

Supplementary table 5: Change in scores of aligned question pairs from pre-use to post-use for whole cohort

Supplementary table 6: Key features of clusters after use of device

Supplementary table 7: Type of sentiment change from Q6 pre-text to Q4 post-text (willingness to wear the device short versus long-term domain), stratified by cluster

Legend for Supplementary figures

Supplementary Figure 1: Representative word clouds based on participants' responses to questionnaires

This figure presents word clouds summarizing participant responses. Panel (a) visualizes responses to the pre-use questionnaire item: "Do you think a monitor that continuously records your baby's heartbeat over days or weeks would make you feel more or less reassured?" Panel (b) displays responses to "What features would you want a continuous baby heart rate monitor to have?" Panel (c) represents responses to the post-use questionnaire item: "Do you prefer the new fetal heartbeat device compared to the existing CTG device? Please tell us why?" Panel (d) illustrates responses to "What features would you want a continuous baby heart monitor to have?"

Supplementary Figure 2: Clustering of participants based on responses to pre-use questionnaires

This figure displays the distribution of participant clusters based on key demographic and clinical features derived from pre-use questionnaire responses.

Supplementary Figure 3: Distribution of binarized response scores of post-use questionnaire responses

Supplementary Figure 4: Cluster Analysis Based on Participant Responses to Pre-Use Q4 (Continuous Use) and Q7 (Home Use)

This figure shows the distribution of Likert scores for the structured components of these questions across clusters. It also depicts the distribution of sentiment scores for unstructured text responses related to these questions.
